# Long-term efficacy of different procedures for treatment of varicose veins: A network meta-analysis

**DOI:** 10.1097/MD.0000000000014495

**Published:** 2019-02-15

**Authors:** Liqin Guo, Rong Huang, Dunyong Zhao, Guilian Xu, Hui Liu, Jian Yang, Tao Guo

**Affiliations:** a School of Nursing, Huanggang Polytechnic College, Huanggang; b Institute of Digestive, Southwest Hospital, Third Military Medical University, Chongqing; c Department of Integrated Science, Huanggang Maternal and Child Health-Care Hospital, Huanggang; d Department of Hepatobiliary and Pancreatic Surgery, Zhongnan Hospital of Wuhan University, Wuhan, China.

**Keywords:** long-term efficacy, network meta-analysis, varicose veins

## Abstract

**Background:**

Various procedures for the treatment of varicose veins have been shown to have long-term effectiveness, but research has yet to identify the most effective procedure. The aim of this study was to investigate the long-term efficacy of different procedures based on Bayesian network meta-analysis and to rank therapeutic options for clinical decision-making.

**Methods:**

Globally recognized databases, namely, MEDLINE, Embase, and Cochrane Central, were searched for randomized controlled trials (RCTs). Quantitative pooled estimation of successful treatment rate (STR) and recurrence rate (RR) was performed to assess the long-term efficacy of each procedure with more than a 1-year follow-up. The surface under the cumulative ranking (SUCRA) probabilities of the *P* values regarding STR and RR were calculated to rank various procedures. Grades of Recommendations Assessment, Development and Evaluation (GRADE) criteria were utilized for the recommendation of evidence from pairwise direct comparisons.

**Results:**

A total of 39 RCTs encompassing a total of 6917 limbs were eligible and provided relative raw data. After quantitative analysis, the CHIVA procedure was determined to have the best long-term efficacy, as it had the highest STR (SUCRA, 0.37). Additionally, the results revealed that CHIVA possessed the highest probability of achieving the lowest long-term RR (SUCRA, 0.61). Moreover, the sensitivity analysis with inconsistency approach clarified the reliability of the main results, and the evidence of most direct comparisons was ranked as high or moderate.

**Conclusion:**

CHIVA seemed to have superior clinical benefits on long-term efficacy for treating varicose veins. However, the conclusion still needs additional trials for supporting evidence.

## Abstract 2

Supplemental Digital Content is available in the text

## 1 Introduction

Abnormally dilated veins are called varicose veins. Varicose veins result from chronic venous insufficiency, which is a complex condition whereby the veins do not efficiently return blood from the legs to the heart.^[1]^ Varicose veins are commonly found in the lower limbs and may be seen either as dilated and tortuous veins or as palpable veins under the skin. Varicose veins in the lower extremities are a sign of chronic venous disorder due to valvular incompetence of the superficial venous system, and they are highly prevalent in the world.^[2,3]^ Although varicose veins do not usually cause fatal conditions, concomitant complications will occur,^[4,5]^ and they may significantly reduce the quality of life; therefore, effective therapies are necessary.^[6,7]^

Currently, various approaches and new techniques for the treatment of varicose veins are reported and being developed. For example, the conventional surgical procedure, which includes ligation of incompetent sapheno-popliteal junction or/and stripping of short segment of short saphenous vein, which was applied with variations, is used as a treatment.^[8]^ Despite continuous innovations, this conventional procedure still has a high risk of complications and recurrence.^[9]^ On the other hand, minimally invasive techniques have been developed to treat sapheno-femoral junction and saphenous vein incompetence. These techniques mainly contain ablation (by laser or radiofrequency) and sclerotherapy (by foam or solutions), which were demonstrated to be safe and effective and are now widely used in clinical practice.^[10–12]^ In 1988, Franceschi described a new method called “Ambulatory Conservative Hemodynamic Management of Varicose Veins” (CHIVA), which integrated the advantages of conventional open surgery and minimally invasive endovascular procedures.^[13]^ Previous research has shown that CHIVA decreases the diameter of the saphenous vein and exhibited clinical efficacy.^[14,15]^

In the past decade, more and more randomized controlled trials (RCTs) for long-term efficacy comparing different therapeutic procedures for varicose veins have been published, but the comprehensive quantitative analysis was rarely reported, and the superior procedure remains largely debated. Therefore, in the current study, a comprehensive quantitative Bayesian network meta-analysis was performed to summarize the evidence for better clinical decision-making in the future.

## 2 Methods

Current meta-analysis was based entirely on previous published studies which had declared ethical approvals and no original clinical raw data was collected or utilized, thereby ethical approval was not conducted for this study. This review was conducted using a predefined protocol and was performed in accordance with PRISMA guidelines.^[16]^ Moreover, this review was registered online at the Research Registry Center with obtained UIN number reviewregistry537.

### 2.1 Inclusion and exclusion criteria

Inclusion criteria were as follows: randomized controlled trials (RCTs) that compared different procedures; sufficient raw data of long-term outcomes; follow-up period ≥ 1 year; English-language titles or abstracts must be located in abovementioned databases.

Exclusion criteria were as follows: retrospective, cohort or observational studies; raw data was not available; short-term clinical trials with follow-up < 1 year; basic science research; any reviews or comments; the full English text could not be traced; trials of comparisons between same procedures or mixed procedures.

### 2.2 Identification and selection of studies

We searched for studies that were published prior to May 15, 2018 in 3 globally recognized databases, namely, MEDLINE, Embase, and Cochrane Central Register of Controlled Trials. Relative MeSH and items were separated and combined for comprehensive identification (MEDLINE search strategy example and details searched were available in Supplementary Table S1). The full English texts were obtained without restriction of publication status. According to abovementioned criteria, trials that compared various procedures were independently reviewed by 2 investigators for final inclusions. Any controversies were resolved by group discussion.

### 2.3 Data extraction and outcome of interest

In the current study, the focus was on long-term efficacy of different procedures to treat varicose veins. Thus, the objective data, namely, successful treatment rate (STR) and recurrence rate (RR), were chosen for the final data synthesis. STR was defined as anatomic and functional completeness, with completely ablated, occluded, or stripped by ultrasound confirmation. Clinical RR was regarded as any visual varicose or reflux confirmations after initial procedure.

For data extraction, the raw data of STR and RR at each year were independently extracted by 2 reviewers for analysis. General information (e.g., author names, publication years) was also collected for detailed presentation. In addition, for those papers presenting only survival curves, Engauge Digitizer software (version 4.1) was used to extract raw data of STR or RR,^[17,18]^ and any debates of data appraisal were resolved by group discussion.

### 2.4 Quality assessment and recommendation of evidence

The Cochrane Risk of Bias assessment tool^[19]^ was used to assess the quality of included trials based on 6 risk factors of bias (selection, performance, detection, attrition, reporting, and others). Each trial was evaluated, and the details of the overall bias risk would be presented. To further confirm the reliability of current study, the Grades of Recommendations Assessment, Development and Evaluation (GRADE) criteria were selected to assess the methodological quality of the evidence.^[20]^ The GRADE criteria include 8 items to comprehensively judge the recommendation of evidence. The quality assessment of each trial and the evidence judgement were performed individually by 2 investigators with follow-up group discussion until reaching agreement. A table of the GRADE system was generated by the software GRADE profiler (version 3.6).

### 2.5 Statistical analysis

Pooled estimation for this network meta-analysis was conducted based on the Bayesian theorem, which considered an extension of the traditional pairwise meta-analysis. This approach incorporates both direct and indirect information through a common comparator to obtain estimates of the relative interventional effects on multiple intervention comparisons.^[21,22]^ The surface under the cumulative ranking (SUCRA) probabilities of the *P* values was presented to clarify the pros and cons of different procedures. That is to say, the accumulative *P* values represented the possibility of achieving the highest STR or the lowest RR after quantitative calculations. Odds ratios (ORs) and 95% confidence intervals (CIs) derived from the network meta-analysis were calculated to exhibit the comparison of different interventions, and publication bias was assessed by examining funnel plots.

Sensitivity analyses were performed based on the inconsistency calculation model. This approach was used to test the consistency of the main results, based on the node-splitting analysis.^[23]^*P* > 0.05 indicates that there is no statistical inconsistency. Data analyses were conducted using the Stata software package (version 12.0),^[24]^ and the data model was verified by using the automated software Aggregate Data Drug Information System (ADDIS, version 1.16).

## 3 Results

### 3.1 Study characteristics and quality assessment

**Figure 1:**
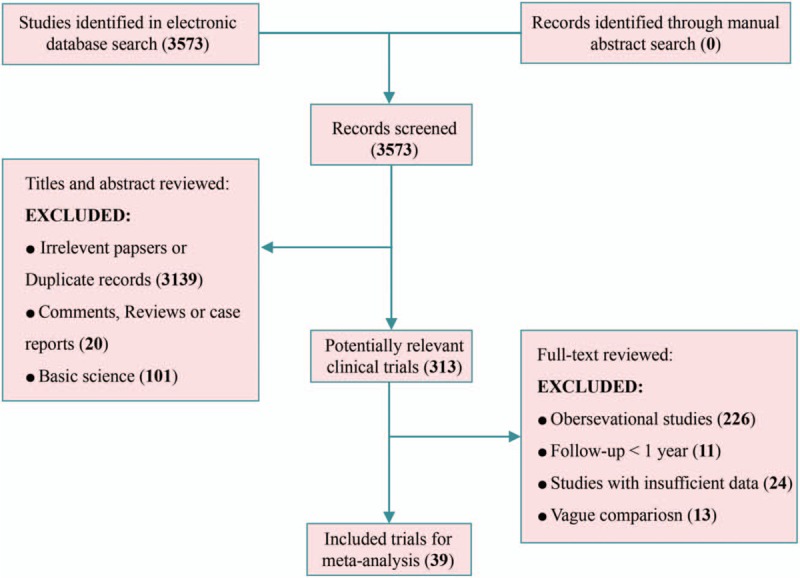
Flow diagram of the process of (and the reasons for) including and excluding studies for this meta-analysis.

**Figure 2:**
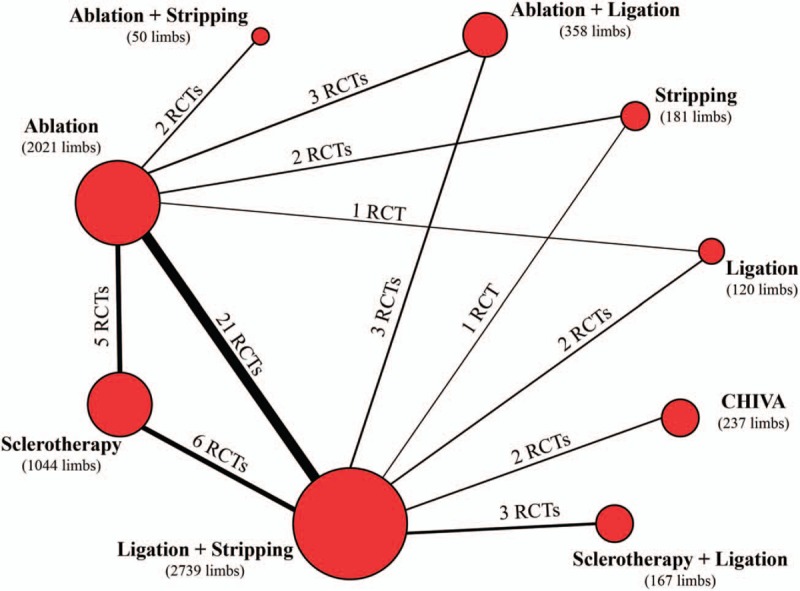
Network connections of all included trails. The numbers on the line indicate the quality of studies compared with every pair of procedures, which were also represented by the width of the lines. Additionally, the sizes of the areas of the circles stand for the respective sample sizes.

After identifying 3573 relative records, 39 RCTs containing 6917 limbs were included in the network meta-analysis (Fig. 1). The included studies were published between 1974 and 2017 and included 1- to 10-year follow-ups, and most of the included studies were performed in European countries (32/39). In summary, 5 procedures, namely, ablation, CHIVA, sclerotherapy, ligation, and stripping, as well as 4 additional combination treatments (ablation plus stripping, ablation plus ligation, sclerotherapy plus ligation, ligation plus stripping) were quantitatively analyzed (details in Supplementary Table S2). The network plot of all included catalogs and available direct connections is shown in Figure 2.

Regarding quality assessment, 19 trials (48%) reported random sequence generation with concealed allocation. However, the application of blinding methods was rarely reported (details in supplementary Figure 1). Thus, the included studies were considered to have a high risk of bias.

### 3.2 The results of network meta-analysis

**Figure 3:**
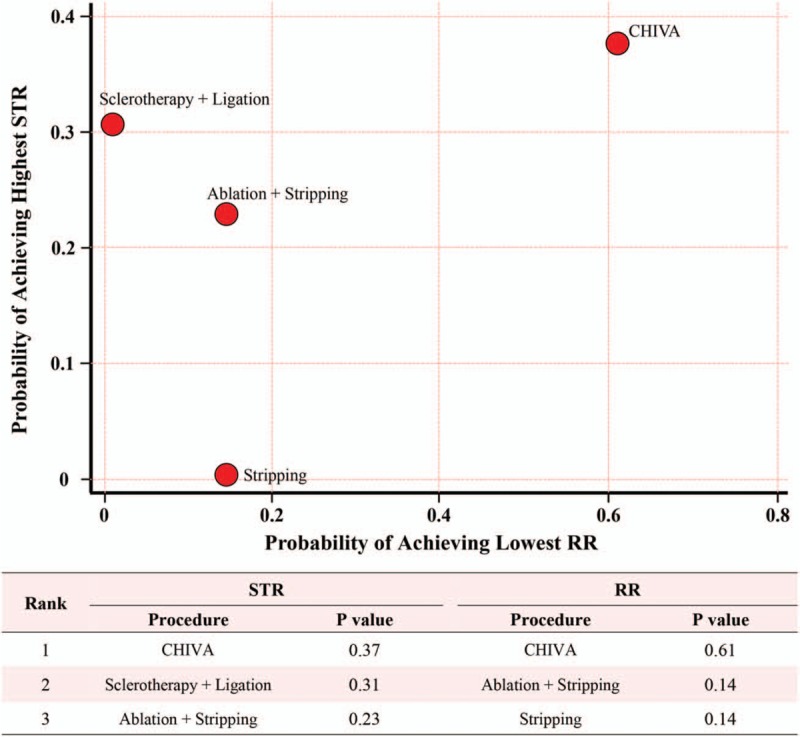
Scatter plot of surface under the cumulative ranking curve values of top 3 procedures regarding successful treatment rate and recurrence rate. The specific top 3 *P* values were also presented beneath the plot.

For the long-term outcome of STR, 24 trials encompassing a total of 4424 limbs contained relative data and head-to-head comparisons between the different procedures were depicted as network plots (Supplementary Figure S2A). After pooled estimation, network odds ratios (ORs) and relative 95% CIs for each possible comparison of 9 procedures were presented (Supplementary Table S3). The results indicated that CHIVA seemed to be associated with higher odds of STR when compared to the other procedures. Furthermore, CHIVA had the highest STR with the highest cumulative *P* values (SUCRA, 0.37), followed by sclerotherapy plus ligation (SUCRA, 0.31) and ablation plus stripping (SUCRA, 0.23) (Fig. 3).

Meanwhile, for the long-term recurrence rate (RR), 30 trials encompassing a total of 5039 limbs provided the raw data, and the network plot presented the available direct comparison (exhibited in Supplementary Figure S2B). Interestingly, based on network meta-analysis, CHIVA was associated with lower odds of RR compared to the other procedures (Supplementary Table S3). Similarly, CHIVA had the highest probability of achieving the lowest RR (SUCRA, 0.61), followed by ablation plus stripping (SUCRA, 0.14) and stripping (SUCRA, 0.14) (Fig. 3).

### 3.3 Sensitivity analysis and data consistency test

To ensure the reliability and consistency of the results, sensitivity analysis was performed using the inconsistency calculation approach. The relative ORs were estimated and presented in detail (Supplementary Table S4). Overall, the results of the sensitivity analysis were similar to the main outcomes. To further clarify the network coherence and data consistency at a statistical level, the differences between the direct and indirect effects in the closed loops were estimated by a node-splitting model. The results exhibited no data inconsistency existed regarding either STR or RR (*P* > 0.05 for all) (Supplementary Table S5).

### 3.4 Publication bias and quality of evidence

According to the funnel plots, no obvious publication bias was detected in STR (Supplementary Figure S3A) and RR (Supplementary Figure S3B). However, 11 direct comparisons for STR and 12 for RR were summed for the judgement of evidence according to GRADE criteria. It was proven that most direct evidence was rated as moderate or high quality (Supplementary Table S6).

## 4 Discussion

In the current study, the aim is to discover the long-term efficacy of various procedures for the treatment of varicose veins by comparing STR and RR with more than a 1-year follow-up. Included in the study were 39 RCTs encompassing a total of 6917 limbs for final quantitative analysis. Among them, 5 procedures and 4 combination treatments were classified for direct and indirect comparisons. After quantitative analysis of successful treatment rate, the study proved that CHIVA had the highest STR in more than a 1-year follow-up (cumulative *P*, 0.37). Similarly, in an analysis for the recurrence rate after a 1-year follow-up, the results revealed that CHIVA had the lowest RR (cumulative *P*, 0.61). In addition, sensitivity analysis clarified the reliability and consistency of the main results, based on the inconsistency approach and the node-splitting model. However, although no obvious publication bias was detected according to funnel plots, a quality analysis revealed that a high risk of bias may exist, and most evidence was rated as moderate.

According to the results, CHIVA seemed to have superior long-term clinical efficacy compared with other procedures. The aim of CHIVA is not only to preserve the great saphenous vein for use as a future vascular graft but also to maintain its drainage eliminating reflux points with change of compartments.^[25–27]^ In practice, the CHIVA method consists of breaking up the hydrostatic pressure column by disconnecting venous shunts. Varicose veins decrease in diameter while continuing to serve their function draining to the deep venous system due to the fragmentation of the HPC and the suction effect of the valvulomuscular pump.^[28,29]^ This revolutionary subverted conventional treatment ideas focused on the invasive removal or destruction of nonfunctional saphenous veins, whereas this approach may be more suitable for reserving the normal physiological process and could be performed safely. Facts also proved that CHIVA showed better results in the duplex imaging and safety variables (e.g., postoperative side effects in convalescent time).^[9,30]^ Conversely, conventional ligation plus stripping was the golden standard for the treatment of varicose veins previously. It may improve the quality of life and reveal good efficacy during short-term postoperative period. Nevertheless, conventional surgery could not prevent varicose vein recurrence or remodeling of the venous network of subcutaneous tissue, thus, it may not maintain long-term clinical benefit.^[31–33]^ This deduction seemed to be consistent with a previous small sample meta-analysis.^[34]^ Moreover, some minimally invasive techniques such as ablation and sclerotherapy were also considered effective.^[35–37]^ Ablation or sclerotherapy may bring less trauma and therefore, could be safer than the conventional surgical procedure. More importantly, minimally invasive techniques also showed better long-term effectiveness compared to conventional procedures.^[38,39]^ Minimally invasive techniques may be a better substitution for conventional surgery in order to be the superior procedure. However, despite less trauma and higher efficacy, ablation and sclerotherapy were still invasive treatments that could also cause physiological destruction and complications. In addition, for long-term follow-up, minimally invasive techniques could not fundamentally prevent varicose vein recurrence or remodeling of the venous network. However, the revolution of CHIVA is to avoid these problems based on initial physical process by preserving saphenous vein and disconnecting venous shunts simultaneously. It is understood that varicose veins are an obstacle to the transport of blood, rather than a complete graft damage. Although valve functional injury may be irreversible, blood vessels can still play a role in transport. CHIVA uses venous shunt to reduce the pressure of varicose veins while taking part of the blood flow by using the reduced diameter varicose veins. This new balance may further solve the cause of the varicosity and avoid invasive destruction. These characteristics may be the key factors revealing better clinical long-term efficacy.

To our knowledge, the current study is the first to comprehensively analyze various procedures for the treatment of varicose veins. It was clarified that CHIVA exhibited superior clinical efficacy regarding the parametric data of STR and RR. Theoretically, it was deduced that the CHIVA approach was more suitable for functional recovery and normal physiological process. This may be the key factor influencing long-term outcomes and the revolutionary manifestation. However, after illustration, some shortcomings should be addressed. First, although included in the study were 39 RCTs containing large sample size (6917 limbs), some certain study arms contained fewer participants (only 50 limbs for ablation plus stripping, for instance). Moreover, CHIVA was determined to have superior clinical efficacy compared to other procedures; however, it also contains its drawbacks. For example, CHIVA has higher standards for surgeons’ experience and if performed incorrectly, results were far worse than stripping.^[40]^ Moreover, only 2 relative RCTs were included, and more trials about CHIVA were needed to support the conclusion. Second, the reliability of the results were demonstrated, but most of the included trials were performed in Europe, which may induce a local bias. Meanwhile, the time range of long-term follow-up contained different annual nodes, which may be potential confounding factors. Lastly, network meta-analysis was based on indirect comparisons and ranked by SUCRA probabilities, and due to the scarcity of trails and raw data in current study, pair-wised meta-analysis and indirect safety analysis could not be undertaken. Thus, we draw our conclusions with cautions and more trials should be conducted in the future.

Despite the existence of several limitations, the final conclusions showed that the long-term efficacy of CHIVA was superior to the efficacy of other procedures. The efficacy of this approach was based on a better physiological process, and this revolutionary approach should be widely applied in clinics. However, the conclusion still needs more trials for support.

## Author contributions

**Conceptualization:** Guilian Xu, Hui Liu.

**Data curation:** Hui Liu.

**Investigation:** Liqin Guo, Rong Huang, Hui Liu.

**Methodology:** Liqin Guo, Jian Yang.

**Project administration:** Liqin Guo.

**Resources:** Dunyong Zhao, Guilian Xu.

**Software:** Rong Huang, Dunyong Zhao, Guilian Xu, Jian Yang.

**Supervision:** Rong Huang.

**Validation:** Dunyong Zhao, Tao Guo.

**Visualization:** Tao Guo.

**Writing – original draft:** Liqin Guo, Tao Guo.

**Writing – review & editing:** Tao Guo.

## Supplementary Material

Supplemental Digital Content
