# Supplementary material for: Long-term efficacy of different procedures for treatment of varicose veins: A network meta-analysis
Source: Medicine (Baltimore). 2019 Feb 15;98(7):e14495. doi: 10.1097/MD.0000000000014495 (PMC6408126; doi:10.1097/MD.0000000000014495)
Supplement: Supplemental Digital Content [file medi-98-e14495-s001.pdf]

Supplementary Tables and Figures

Table S1. Example retrieval strategy in MEDLINE..

# No.	Search	Results
1	exp (varix) OR (varicosity) OR (varicose)/	22569
2	exp (cure rate) OR (succeessful treatment)/	271766
3	exp (recurrence) OR (reflux)/	486976
4	2 OR 3	736181
5	1 AND 4	2935
6	(ablation or frequency or thermal or laser). ti,ab.	3299439
7	(sclerotherapy or foam or sclerosing or cyanoacrylate). ti,ab.	49221
8	(ligation). ti,ab.	75866
9	(phlebectomy or stripping). ti,ab.	11716
10	(haemodynamic correction or CHIVA or (Ambulatory Conservative Haemodynamic Management of Varicose Veins). ti,ab.	6904
11	6 OR 7 OR 8 OR 9 OR 10	3425160
12	5 AND 11	1673
13	(randomized controlled trial or prospective). ti,ab.	1323708
14	12 AND 13	1337

Table S2. General information about the included 39 RCTs. ["★" represents available data, while "☆" means not available.]

Author	Country	Year	Study arms	Intervention	Sample size	Follow-up time	Available data	
							STR	RR
Biemans [1]	Netherlands	2012	3	Ablation vs. Sclerotherapy vs. Ligation + Stripping	223	1 year	★	☆
Carandina [2]	Italy	2008	2	CHIVA vs. Ligation + Stripping	124	10 years	☆	★
Carradice [3]	UK	2009	2	Ablation + Stripping vs. Ablation	50	1 year	★	☆
Carradice [4]	UK	2011	2	Ablation vs. Ligation + Stripping	237	1 year	☆	★
Casoni [5]	France	2012	2	Ligation + Stripping vs. Stripping	123	8 year	☆	★
Christenson [6]	Switzerland	2010	2	Ablation vs. Ligation + Stripping	197	2 years	★	☆
Disselhoff [7]	Netherlands	2008	2	Ablation vs. Stripping	120	2 years	☆	★
Disselhoff [8]	Netherlands	2008	2	Ablation vs. Ablation + Ligation	86	2 years	★	★
Disselhoff [9]	Netherlands	2010	2	Ablation vs. Ablation + Ligation	86	5 years	★	★
Disselhoff [10]	Netherlands	2011	2	Ablation vs. Stripping	120	5 years	★	☆
Dwerryhouse [11]	UK	1999	2	Ligation vs. Ligation + Stripping	78	5 years	★	★
ElKaffas [12]	Egypt	2011	2	Ablation vs. Ligation + Stripping	180	2 years	☆	★
El-Sheikha [13]	UK	2014	2	Ablation + Stripping vs. Ablation	50	5 years	☆	★
Flessenkamper [14]	Germany	2016	3	Ablation vs. Ablation + Ligation vs. Ligation + Stripping	442	6 years	☆	★
Gauw [15]	Netherlands	2015	2	Ablation vs. Ligation + Stripping	130	5 years	☆	★
Hobbs [16]	USA	1974	2	Sclerotherapy vs. Ligation + Stripping	679	6 years	★	☆
Kalodiki [17]	UK	2011	2	Sclerotherapy + Ligation vs. Ligation + Stripping	59	5 years	★	★
Kalteis [18]	Austria	2015	2	Ablation + Ligation vs. Ligation + Stripping	72	5 years	★	★
Kolluri [19]	USA	2016	2	Sclerotherapy vs. Ablation	212	1 year	★	☆
Lurie [20]	Austria	2005	2	Ablation vs. Ligation + Stripping	65	2 years	☆	★
Mendes [21]	Brazil	2016	2	Ablation vs. Ligation + Stripping	20	1 year	★	☆
Mozafar [22]	Iran	2014	2	Ablation vs. Ligation	65	18 months	★	★
Nandhra [23]	UK	2015	2	Ablation vs. Ligation + Stripping	106	2 years	★	★
Pare´s [24]	Spain	2010	2	CHIVA vs. Ligation + Stripping	521	5 years	★	★
Perala [25]	Finland	2005	2	Ablation vs. Ligation + Stripping	28	3 years	☆	★
Pronk [26]	Netherlands	2010	2	Ablation vs. Ligation + Stripping	130	1 year	☆	★
Rasmussen [27]	Denmark	2010	2	Ablation vs. Ligation + Stripping	137	2 years	★	★
Rasmussen [28]	Denmark	2011	3	Ablation vs. Sclerotherapy vs. Ligation + Stripping	417	1 year	★	★
Rasmussen [29]	Denmark	2013	2	Ablation vs. Ligation + Stripping	134	5 years	★	★
Rass [30]	Germany	2012	2	Ablation vs. Ligation + Stripping	316	2 years	★	★
Rass [31]	Germany	2015	2	Ablation vs. Ligation + Stripping	281	5 years	★	★
Rutgers [32]	Netherlands	1994	2	Sclerotherapy + Ligation vs. Ligation + Stripping	142	3 years	☆	★
Samuel [33]	UK	2013	2	Ablation vs. Ligation + Stripping	99	1 year	★	★
Sarin [34]	UK	1994	2	Ligation vs. Ligation + Stripping	89	21 months	★	★
Shadid [35]	Netherlands	2012	2	Sclerotherapy vs. Ligation + Stripping	390	2 years	☆	★

Velden [36]	Belgium	2015	3	Ablation vs. Sclerotherapy vs. Ligation + Stripping	193	5 years	☆	★
Venermo [37]	Finland	2016	3	Ablation vs. Sclerotherapy vs. Ligation + Stripping	206	1 year	★	☆
Yang [38]	China	2013	2	Ablation + Ligation vs. Ligation + Stripping	171	2 years	★	☆
Yin [39]	China	2017	2	Sclerotherapy + Ligation vs. Ligation + Stripping	139	1 year	☆	★

STR: successful treatment rate; RR: recurrence rate.

Reference

[1] Biemans AA, Kockaert M, Akkersdijk GP, ET AL. Comparing endovenous laser ablation, foam sclerotherapy, and conventional surgery for great saphenous varicose veins. J Vasc Surg. 2013;58(3):727-34.

[2] Carandina S, Mari C, De Palma M, ET AL. Varicose vein stripping vs haemodynamic correction (CHIVA): a long term randomised trial. Eur J Vasc Endovasc Surg. 2008;35(2):230-7.

[3] Carradice D, Mekako AI, Hatfield J, ET AL. Randomized clinical trial of concomitant or sequential phlebectomy after endovenous laser therapy for varicose veins. Br J Surg. 2009;96(4):369-75.

[4] Carradice D, Mekako AI, Mazari FA, et al. Clinical and technical outcomes from a randomized clinical trial of endovenous laser ablation compared with conventional surgery for great saphenous varicose veins. Br J Surg. 2011;98(8):1117-23.

[5] Casoni P, Lefebvre-Vilardebo M, Villa F, et al. Great saphenous vein surgery without high ligation of the saphenofemoral junction. J Vasc Surg. 2013;58(1):173-8.

[6] Christenson JT, Gueddi S, Gemayel G, et al. Prospective randomized trial comparing endovenous laser ablation and surgery for treatment of primary great saphenous varicose veins with a 2-year follow-up. J Vasc Surg. 2010;52(5):1234-41.

[7] Disselhoff BC, der Kinderen DJ, Kelder JC, et al. Randomized clinical trial comparing endovenous laser ablation of the great Saphenous vein with and without ligation of the sapheno-femoral junction: 2-year results. Eur J Vasc Endovasc Surg. 2008;36(6):713-8.

[8] Disselhoff BC, der Kinderen DJ, Kelder JC, et al. Randomized clinical trial comparing endovenous laser with cryostripping for great saphenous varicose veins. Br J Surg. 2008;95(10):1232-8.

[9] Disselhoff BC, der Kinderen DJ, Kelder JC, et al. Five-year results of a randomised clinical trial of endovenous laser ablation of the great saphenous vein with and without ligation of the saphenofemoral junction. Eur J Vasc Endovasc Surg. 2011;41(5):685-90.

[10] Disselhoff BC, der Kinderen DJ, Kelder JC, et al. Five-year results of a randomized clinical trial comparing endovenous laser ablation with cryostripping for great saphenous varicose veins. Br J Surg. 2011;98(8):1107-11.

[11] Dwerryhouse S, Davies B, Harradine K, et al. Stripping the long saphenous vein reduces the rate of reoperation for recurrent varicose veins: five-year results of a randomized trial. J Vasc Surg. 1999;29(4):589-92.

[12] Helmy ElKaffas K, ElKashef O, ElBaz W. Great saphenous vein radiofrequency ablation versus standard stripping in the management of primary varicose veins-a randomized clinical trial. Angiology. 2011;62(1):49-54.

[13] El-Sheikha J, Nandhra S, Carradice D, et al. Clinical outcomes and quality of life 5 years after a randomized trial of concomitant or sequential phlebectomy following endovenous laser ablation for varicose veins. Br J Surg. 2014;101(9):1093-7.

[14] Flessenkämper I, Hartmann M2, Hartmann K, et al. Endovenous laser ablation with and without high ligation compared to high ligation and stripping for treatment of great saphenous varicose veins: Results of a multicentre randomised controlled trial with up to 6 years follow-up. Phlebology. 2016;31(1):23-33.

[15] Gauw SA, Lawson JA, van Vlijmen-van Keulen CJ, et al. Five-year follow-up of a randomized, controlled trial comparing saphenofemoral ligation and stripping of the great saphenous vein with endovenous laser ablation (980 nm) using local tumescent anesthesia. J Vasc Surg. 2016;63(2):420-8.

[16] Hobbs JT. Surgery and sclerotherapy in the treatment of varicose veins. A random trial. Arch Surg. 1974;109(6):793-6.

[17] Kalodiki E, Lattimer CR, Azzam M, et al. Long-term results of a randomized controlled trial on ultrasound-guided foam sclerotherapy combined with saphenofemoral ligation vs standard surgery for varicose veins. J Vasc Surg. 2012;55(2):451-7.

[18] Kalteis M, Adelsgruber P, Messie-Werndl S, et al. Five-year results of a randomized controlled trial comparing high ligation combined with endovenous laser ablation and stripping of the great saphenous vein. Dermatol Surg. 2015;41(5):579-86.

[19] Kolluri R, Gibson K, Cher D, et al. Roll-in phase analysis of clinical study of cyanoacrylate closure for incompetent great saphenous veins. J Vasc Surg Venous Lymphat Disord. 2016;4(4):407-15.

[20] Lurie F, Creton D, Eklof B, et al. Prospective randomised study of endovenous radiofrequency obliteration (closure) versus ligation and vein stripping (EVOLVeS): two-year follow-up. Eur J Vasc Endovasc Surg. 2005;29(1):67-73.

[21] Mendes CA, Martins AA, Fukuda JM, et al. Randomized trial of radiofrequency ablation versus conventional surgery for superficial venous insufficiency: if you don't tell, they won't know. Clinics (Sao Paulo). 2016;71(11):650-656.

[22] Mozafar M, Atqiaee K, Haghighatkhah H, et al. Endovenous laser ablation of the great saphenous vein versus high ligation: long-term results. Lasers Med Sci. 2014;29(2):765-71.

[23] Nandhra S, El-sheikha J, Carradice D, et al. A randomized clinical trial of endovenous laser ablation versus conventional surgery for small saphenous varicose veins. J Vasc Surg. 2015;61(3):741-6.

[24] Parés JO, Juan J, Tellez R, et al. Varicose vein surgery: stripping versus the CHIVA method: a randomized controlled trial. Ann Surg. 2010;251(4):624-31.

[25] Perälä J, Rautio T, Biancari F, et al. Radiofrequency endovenous obliteration versus stripping of the long saphenous vein in the management of primary varicose veins: 3-year outcome of a randomized study. Ann Vasc Surg. 2005;19(5):669-72.

[26] Pronk P, Gauw SA, Mooij MC, et al. Randomised controlled trial comparing sapheno-femoral ligation and stripping of the great saphenous vein with endovenous laser ablation (980 nm) using local tumescent anaesthesia: one year results. Eur J Vasc Endovasc Surg. 2010;40(5):649-56.

[27] Rasmussen LH, Bjoern L, Lawaetz M, Randomised clinical trial comparing endovenous laser ablation with stripping of the great saphenous vein: clinical outcome and recurrence after 2 years. Eur J Vasc Endovasc Surg. 2010;39(5):630-5.

[28] Rasmussen LH, Lawaetz M, Bjoern L, et al. Randomized clinical trial comparing endovenous laser ablation, radiofrequency ablation, foam sclerotherapy and surgical stripping for great saphenous varicose veins. Br J Surg. 2011;98(8):1079-87.

[29] Rasmussen L, Lawaetz M, Bjoern L, et al. Randomized clinical trial comparing endovenous laser ablation and stripping of the great saphenous vein with clinical and duplex outcome after 5 years. J Vasc Surg. 2013;58(2):421-6.

[30] Rass K, Frings N, Glowacki P, et al. Comparable effectiveness of endovenous laser ablation and high ligation with stripping of the great saphenous vein: two-year results of a randomized clinical trial (RELACS study). Arch Dermatol. 2012;148(1):49-58.

[31] Rass K, Frings N, Glowacki P, et al. Same Site Recurrence is More Frequent After Endovenous Laser Ablation Compared with High Ligation and Stripping of the Great Saphenous Vein: 5 year Results of a Randomized Clinical Trial (RELACS Study). Eur J Vasc Endovasc Surg. 2015;50(5):648-56.

[32] Rutgers PH, Kitslaar PJ. Randomized trial of stripping versus high ligation combined with sclerotherapy in the treatment of the incompetent greater saphenous vein. Am J Surg. 1994;168(4):311-5.

[33] Samuel N, Carradice D, Wallace T, et al. Randomized clinical trial of endovenous laser ablation versus conventional surgery for small saphenous varicose veins. Ann Surg. 2013;257(3):419-26.

[34] Sarin S, Scurr JH, Coleridge Smith PD. Stripping of the long saphenous vein in the treatment of primary varicose veins. Br J Surg. 1994;81(10):1455-8.

[35] Shadid N, Ceulen R, Nelemans P, et al. Randomized clinical trial of ultrasound-guided foam sclerotherapy versus surgery for the incompetent great saphenous vein. Br J Surg. 2012;99(8):1062-70.

[36] Van der Velden SK, Biemans AA, De Maeseneer MG, et al. Five-year results of a randomized clinical trial of conventional surgery, endovenous laser ablation and ultrasound-guided foam sclerotherapy in patients with great saphenous varicose veins. Br J Surg. 2015;102(10):1184-94.

[37] Venermo M, Saarinen J, Eskelinen E, et al. Randomized clinical trial comparing surgery, endovenous laser ablation and ultrasound-guided foam sclerotherapy for the treatment of great saphenous varicose veins. Br J Surg. 2016;103(11):1438-44.

[38] Yang L, Wang XP, Su WJ, et al. Randomized clinical trial of endovenous microwave ablation combined with high ligation versus conventional surgery for varicose veins. Eur J Vasc Endovasc Surg. 2013;46(4):473-9.

[39] Yin H, He H, Wang M, et al. Prospective Randomized Study of Ultrasound-Guided Foam Sclerotherapy Combined with Great Saphenous Vein High Ligation in the Treatment of Severe Lower Extremity Varicosis. Ann Vasc Surg. 2017;39:256-263.

Table S3. Network comparison of various procedures with long-term STR and RR.

Comparisons [Odds ratio (95% credible intervals)]									
**Regarding STR**	Ablation	2.12 (0.40, 12.42)	2.63 (0.06, 206.45)	7.04 (0.41, 128.79)	0.93 (0.15, 6.32)	2.75 (1.22, 7.25)	0.46 (0.14, 1.77)	5.41 (0.29, 123.18)	0.27 (0.01, 4.91)
	0.47 (0.08, 2.52)	Ablation + Ligation	1.27 (0.02, 125.86)	3.28 (0.12, 88.35)	0.44 (0.04, 4.72)	1.31 (0.24, 6.93)	0.22 (0.03, 1.68)	2.64 (0.09, 80.51)	0.13 (0.00, 3.61)
	0.38 (0.00, 17.40)	0.79 (0.01, 56.57)	Ablation + Stripping	2.61 (0.02, 358.68)	0.35 (0.00, 26.19)	1.05 (0.01, 55.16)	0.17 (0.00, 10.34)	2.08 (0.01, 294.37)	0.10 (0.00, 12.17)
	0.14 (0.01, 2.43)	0.30 (0.01, 8.35)	0.38 (0.00, 61.64)	CHIVA	0.14 (0.00, 3.35)	0.39 (0.03, 6.09)	0.07 (0.00, 1.40)	0.79 (0.01, 41.17)	0.04 (0.00, 2.05)
	1.08 (0.16, 6.60)	2.26 (0.21, 25.30)	2.89 (0.04, 300.29)	7.40 (0.30, 210.97)	Ligation	2.97 (0.52, 17.34)	0.51 (0.06, 4.12)	5.87 (0.20, 179.34)	0.29 (0.01, 8.09)
	0.36 (0.14, 0.82)	0.76 (0.14, 4.14)	0.95 (0.02, 75.42)	2.53 (0.16, 39.29)	0.34 (0.06, 1.92)	Ligation + Stripping	0.17 (0.05, 0.60)	1.99 (0.11, 38.80)	0.10 (0.00, 1.88)
	2.15 (0.56, 7.37)	4.53 (0.59, 35.67)	5.77 (0.10, 492.94)	14.81 (0.71, 316.61)	1.96 (0.24, 16.64)	5.88 (1.67, 21.45)	Sclerotherapy	11.81 (0.51, 290.74)	0.58 (0.02, 13.31)
	0.18 (0.01, 3.50)	0.38 (0.01, 11.24)	0.48 (0.00, 93.65)	1.27 (0.02, 68.74)	0.17 (0.01, 5.01)	0.50 (0.03, 9.30)	0.08 (0.00, 1.97)	Sclerotherapy + Ligation	0.05 (0.00, 3.40)
	3.68 (0.20, 78.83)	7.93 (0.28, 267.06)	9.85 (0.08, 1804.78)	25.27 (0.49, 1695.57)	3.49 (0.12, 122.22)	10.02 (0.53, 258.80)	1.71 (0.08, 49.19)	20.37 (0.29, 1477.95)	Stripping
**Regarding RR**	Ablation	0.90 (0.37, 2.21)	1.37 (0.13, 14.16)	0.43 (0.12, 1.62)	2.52 (0.76, 8.80)	1.07 (0.68, 1.70)	1.98 (0.75, 5.20)	2.15 (0.66, 6.94)	0.89 (0.22, 3.67)
	1.12 (0.45, 2.71)	Ablation + Ligation	1.52 (0.13, 18.23)	0.48 (0.10, 2.28)	2.80 (0.65, 12.55)	1.20 (0.49, 2.97)	2.19 (0.62, 8.10)	2.39 (0.57, 9.69)	0.99 (0.19, 5.27)
	0.73 (0.07, 7.50)	0.66 (0.05, 7.74)	Ablation + Stripping	0.31 (0.02, 4.56)	1.85 (0.13, 24.03)	0.79 (0.07, 7.99)	1.46 (0.11, 17.42)	1.56 (0.12, 19.59)	0.67 (0.04, 9.38)
	2.33 (0.62, 8.68)	2.09 (0.44, 9.63)	3.22 (0.22, 42.32)	CHIVA	5.94 (1.06, 31.75)	2.51 (0.72, 8.61)	4.62 (0.91, 21.04)	4.93 (0.93, 25.57)	2.05 (0.32, 13.40)
	0.40 (0.11, 1.32)	0.36 (0.08, 1.54)	0.54 (0.04, 7.52)	0.17 (0.03, 0.94)	Ligation	0.42 (0.13, 1.38)	0.78 (0.17, 3.54)	0.84 (0.17, 4.03)	0.35 (0.06, 2.16)
	0.93 (0.59, 1.47)	0.84 (0.34, 2.06)	1.27 (0.13, 13.36)	0.40 (0.12, 1.39)	2.36 (0.72, 7.96)	Ligation + Stripping	1.83 (0.70, 4.78)	1.99 (0.66, 5.87)	0.82 (0.20, 3.39)
	0.51 (0.19, 1.34)	0.46 (0.12, 1.61)	0.69 (0.06, 8.96)	0.22 (0.05, 1.09)	1.28 (0.28, 5.90)	0.55 (0.21, 1.43)	Sclerotherapy	1.08 (0.25, 4.63)	0.46 (0.09, 2.45)
	0.46 (0.14, 1.50)	0.42 (0.10, 1.77)	0.64 (0.05, 8.25)	0.20 (0.04, 1.08)	1.19 (0.25, 5.84)	0.50 (0.17, 1.51)	0.93 (0.22, 4.00)	Sclerotherapy + Ligation	0.42 (0.07, 2.48)
	1.13 (0.27, 4.46)	1.01 (0.19, 5.15)	1.50 (0.11, 23.01)	0.49 (0.07, 3.11)	2.87 (0.46, 17.62)	1.21 (0.29, 4.88)	2.19 (0.41, 11.68)	2.40 (0.40, 13.91)	Stripping

In each subtable, column procedure is compared with row procedure in upper right part, and raw procedure is compared with column procedure in lower left part. Summary estimate represents odds ratio of STR or RR in respective subtables. STR: successful treatment rate; RR: recurrence rate.

Table S4. Sensitivity analysis: comparison of various procedures by inconsistency approach.

Comparisons [Odds ratio (95% credible intervals)]									
**Regarding STR**	Ablation	2.41 (0.40, 17.34)	2.61 (0.05, 214.54)	9.65 (0.37, 272.00)	0.90 (0.07, 10.32)	2.73 (1.15, 7.45)	0.46 (0.12, 1.86)	7.55 (0.23, 262.73)	0.27 (0.01, 5.05)
	0.41 (0.06, 2.50)	Ablation + Ligation	1.07 (0.01, 135.79)	3.84 (0.15, 114.15)	0.52 (0.04, 6.83)	1.49 (0.25, 9.50)	0.29 (0.02, 4.23)	2.99 (0.09, 99.22)	0.11 (0.00, 3.59)
	0.38 (0.00, 19.22)	0.93 (0.01, 78.17)	Ablation + Stripping	3.69 (0.02, 710.00)	0.49 (0.00, 53.78)	1.38 (0.01, 112.87)	0.28 (0.00, 30.40)	2.84 (0.01, 591.34)	0.10 (0.00, 13.39)
	0.10 (0.00, 2.73)	0.26 (0.01, 6.69)	0.27 (0.00, 65.67)	CHIVA	0.14 (0.00, 4.38)	0.38 (0.02, 6.66)	0.08 (0.00, 2.62)	0.77 (0.01, 51.86)	0.03 (0.00, 2.60)
	1.11 (0.10, 13.78)	1.92 (0.15, 25.45)	2.04 (0.02, 287.24)	7.34 (0.23, 235.56)	Ligation	2.84 (0.45, 20.26)	0.56 (0.04, 8.56)	5.73 (0.16, 215.18)	0.21 (0.00, 9.18)
	0.37 (0.13, 0.87)	0.67 (0.11, 3.96)	0.73 (0.01, 76.72)	2.63 (0.15, 42.69)	0.35 (0.05, 2.23)	Ligation + Stripping	0.19 (0.03, 1.60)	2.02 (0.09, 39.98)	0.07 (0.00, 2.17)
	2.20 (0.54, 8.33)	3.48 (0.24, 41.61)	3.61 (0.03, 479.86)	13.25 (0.38, 443.47)	1.78 (0.12, 25.50)	5.22 (0.63, 35.18)	Sclerotherapy	10.22 (0.24, 363.38)	0.38 (0.01, 13.16)
	0.13 (0.00, 4.43)	0.33 (0.01, 11.43)	0.35 (0.00, 98.63)	1.30 (0.02, 87.93)	0.17 (0.00, 6.20)	0.50 (0.03, 11.15)	0.10 (0.00, 4.13)	Sclerotherapy + Ligation	0.04 (0.00, 3.94)
	3.71 (0.20, 74.95)	9.09 (0.28, 339.06)	9.93 (0.07, 1884.20)	36.52 (0.39, 3061.95)	4.72 (0.11, 233.69)	13.42 (0.46, 465.24)	2.62 (0.08, 135.61)	27.68 (0.25, 2802.99)	Stripping
**Regarding RR**	Ablation	0.93 (0.38, 2.26)	1.33 (0.14, 12.75)	0.49 (0.10, 2.48)	2.62 (0.52, 13.20)	1.05 (0.67, 1.68)	2.07 (0.74, 5.77)	2.43 (0.58, 10.74)	1.10 (0.24, 6.16)
	1.08 (0.44, 2.62)	Ablation + Ligation	1.45 (0.13, 16.91)	0.53 (0.10, 2.94)	3.15 (0.62, 15.19)	1.32 (0.44, 4.35)	2.08 (0.44, 9.49)	2.63 (0.56, 12.60)	0.91 (0.16, 5.34)
	0.75 (0.08, 7.04)	0.69 (0.06, 7.91)	Ablation + Stripping	0.38 (0.02, 5.62)	2.21 (0.15, 30.81)	0.92 (0.08, 10.51)	1.43 (0.10, 19.82)	1.85 (0.12, 25.34)	0.64 (0.04, 10.23)
	2.03 (0.40, 9.62)	1.87 (0.34, 10.04)	2.66 (0.18, 43.04)	CHIVA	5.76 (1.06, 32.58)	2.47 (0.72, 8.86)	3.86 (0.65, 21.97)	4.93 (0.92, 25.31)	1.69 (0.25, 12.69)
	0.38 (0.08, 1.92)	0.32 (0.07, 1.62)	0.45 (0.03, 6.65)	0.17 (0.03, 0.95)	Ligation	0.42 (0.13, 1.45)	0.66 (0.11, 3.55)	0.85 (0.17, 4.32)	0.29 (0.04, 2.01)
	0.95 (0.59, 1.50)	0.76 (0.23, 2.26)	1.09 (0.10, 12.16)	0.40 (0.11, 1.38)	2.36 (0.69, 7.71)	Ligation + Stripping	1.58 (0.42, 5.21)	1.99 (0.67, 5.80)	0.69 (0.14, 3.30)
	0.48 (0.17, 1.36)	0.48 (0.11, 2.29)	0.70 (0.05, 9.92)	0.26 (0.05, 1.55)	1.51 (0.28, 8.75)	0.63 (0.19, 2.41)	Sclerotherapy	1.28 (0.24, 7.00)	0.44 (0.06, 3.06)
	0.41 (0.09, 1.72)	0.38 (0.08, 1.79)	0.54 (0.04, 8.01)	0.20 (0.04, 1.09)	1.18 (0.23, 5.87)	0.50 (0.17, 1.48)	0.78 (0.14, 4.12)	Sclerotherapy + Ligation	0.35 (0.05, 2.33)
	0.91 (0.16, 4.22)	1.09 (0.19, 6.27)	1.56 (0.10, 24.78)	0.59 (0.08, 4.00)	3.40 (0.50, 23.82)	1.45 (0.30, 7.10)	2.26 (0.33, 15.54)	2.90 (0.43, 19.83)	Stripping

In each subtable, column procedure is compared with row procedure in upper right part, and raw procedure is compared with column procedure in lower left part. Summary estimate represents odds ratio of STR or RR in respective subtables. STR: successful treatment rate; RR: recurrence rate.

Table S5. Results of node-splitting models by testing the direct and indirect effects. Potential inconsistency may exist if P < 0.05.

	Comparison	Direct Effect	Indirect Effect	Overall	P-Value
Regarding STR	Ablation vs. Ablation + Ligation	1.97 (-0.41, 4.77)	-0.33 (-2.70, 2.10)	0.75 (-0.93, 2.52)	0.16
	Ablation vs. Ligation	-0.73 (-4.18, 2.65)	0.25 (-2.04, 2.67)	-0.07 (-1.89, 1.84)	0.63
	Ablation vs. Ligation + Stripping	0.90 (-0.03, 1.92)	1.14 (-0.86, 3.25)	1.01 (0.20, 1.98)	0.81
	Ablation vs. Sclerotherapy	-1.07 (-2.54, 0.50)	-0.14 (-3.11, 2.94)	-0.77 (-2.00, 0.57)	0.54
	Ablation + Ligation vs. Ligation + Stripping	1.17 (-0.95, 3.44)	-1.18 (-3.99, 1.52)	0.27 (-1.42, 1.94)	0.18
	Ligation vs. Ligation + Stripping	0.83 (-1.37, 3.03)	1.79 (-1.70, 5.37)	1.09 (-0.65, 2.85)	0.62
	Ligation + Stripping vs. Sclerotherapy	-1.77 (-3.07, -0.44)	1.06 (-2.37, 5.07)	-1.77 (-3.07, -0.51)	0.13
Regarding RR	Ablation vs. Ablation + Ligation	-0.09 (-1.17, 0.99)	-0.15 (-2.10, 1.81)	-0.11 (-1.00, 0.79)	0.95
	Ablation vs. Ligation	0.77 (-1.65, 3.50)	1.01 (-0.43, 2.48)	0.92 (-0.28, 2.18)	0.86
	Ablation vs. Ligation + Stripping	-0.00 (-0.47, 0.48)	0.47 (-0.71, 1.67)	0.07 (-0.38, 0.53)	0.45
	Ablation vs. Sclerotherapy	0.79 (-0.44, 2.08)	0.31 (-1.54, 2.12)	0.68 (-0.29, 1.65)	0.65
	Ablation vs. Stripping	0.82 (-1.29, 2.96)	-0.90 (-2.81, 1.00)	-0.12 (-1.49, 1.30)	0.22
	Ablation + Ligation vs. Ligation + Stripping	0.30 (-0.96, 1.57)	0.17 (-1.03, 1.32)	0.18 (-0.72, 1.09)	0.87
	Ligation vs. Ligation + Stripping	-0.94 (-2.29, 0.43)	-0.64 (-3.35, 1.87)	-0.86 (-2.07, 0.32)	0.84
	Ligation + Stripping vs. Stripping	-0.90 (-2.72, 0.91)	0.77 (-1.24, 2.92)	-0.19 (-1.59, 1.22)	0.23

STR: successful treatment rate; RR: recurrence rate.

Table S6. Summary of overall GRADE quality of evidence from direct comparisons.

Comparison		Quality of the evidence (STR)	Quality of the evidence (RR)
Ablation	Ablation + Ligation	⊕ ⊕	⊕ ⊕ ⊕
	Ablation + Stripping	⊕ ⊕	⊕ ⊕
	Ligation	⊕ ⊕	⊕ ⊕
	Ligation + Stripping	⊕ ⊕ ⊕ ⊕	⊕ ⊕ ⊕ ⊕
	Sclerotherapy	⊕ ⊕ ⊕	⊕ ⊕
	Stripping	⊕ ⊕	⊕ ⊕
Ablation + Ligation	Ligation + Stripping	⊕ ⊕	⊕ ⊕
CHIVA	Ligation + Stripping	⊕ ⊕	⊕ ⊕ ⊕
Ligation	Ligation + Stripping	⊕ ⊕	⊕ ⊕
Ligation + Stripping	Sclerotherapy	⊕ ⊕ ⊕	⊕ ⊕
	Sclerotherapy + Ligation	⊕ ⊕	⊕ ⊕ ⊕
	Stripping	—	⊕ ⊕

GRADE Working Group grades of evidence

High quality (⊕ ⊕ ⊕ ⊕): Further research is very unlikely to change our confidence in the estimate of effect.

Moderate quality (⊕ ⊕ ⊕): Further research is likely to have an important impact on our confidence in the estimate of effect and may change the estimate.

Low quality (⊕ ⊕): Further research is very likely to have an important impact on our confidence in the estimate of effect and is likely to change the estimate.

Very low quality (⊕): We are very uncertain about the estimate.

STR: successful treatment rate; RR: recurrence rate.

Figure S1. Quality assessment of **(A)** overall and **(B)** study-level risk of bias, using Cochrane's risk of bias assessment tool.

Figure S1A.

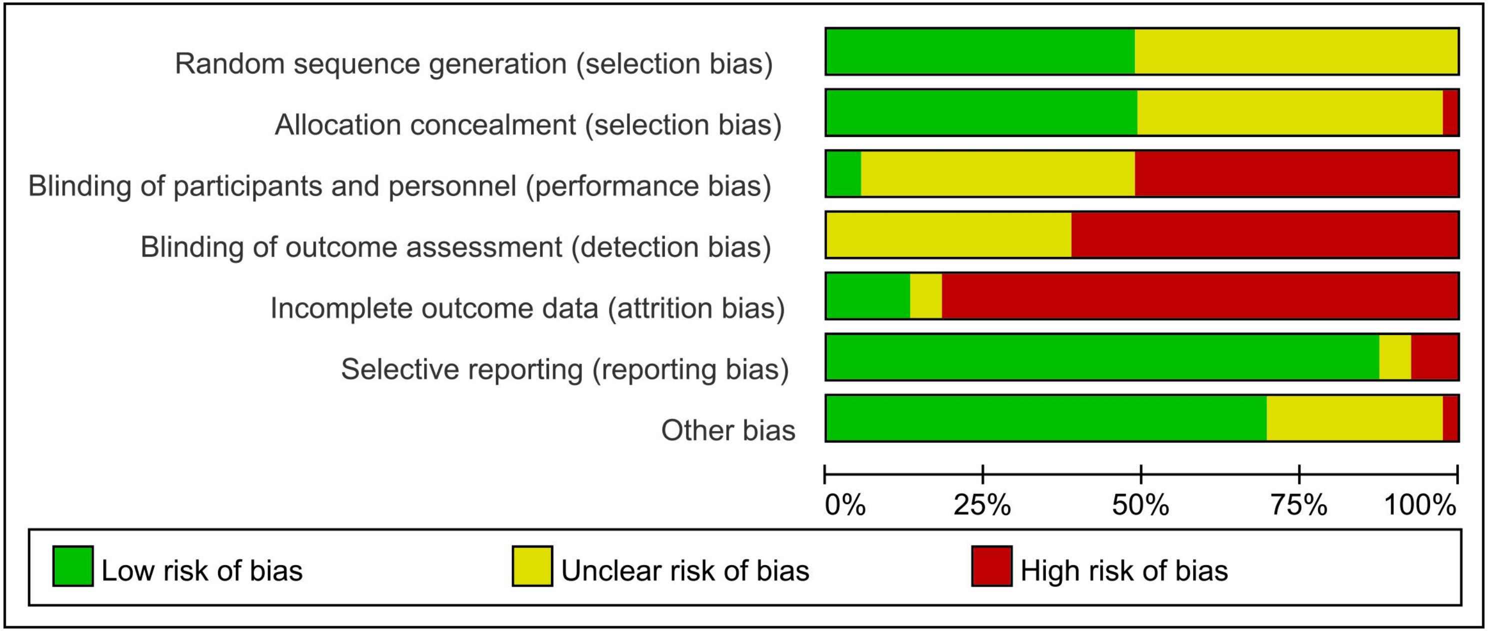

Figure S1B.

	Random sequence generation (selection bias)	Allocation concealment (selection bias)	Blinding of participants and personnel (performance bias)	Blinding of outcome assessment (detection bias)	Incomplete outcome data (attrition bias)	Selective reporting (reporting bias)	Other bias
Biemans 2012	+	−	−	−	−	+	+
Carandina 2008	+	+	−	−	−	+	+
Carradice 2009	?	+	?	?	−	+	?
Carradice 2011	?	?	?	?	−	+	?
Casoni 2012	?	?	?	?	?	+	?
Christenson 2010	?	?	?	?	−	+	+
Disselhoff 2008	?	?	?	?	−	+	?
Disselhoff 2008 (2)	?	?	−	−	−	+	+
Disselhoff 2010	+	+	?	?	−	+	+
Disselhoff 2011	+	+	?	?	−	+	+
Dwerryhouse 1999	?	?	?	?	−	+	+
ElKaffas 2011	?	?	−	−	+	+	+
El-Sheikha 2014	+	+	−	−	−	+	+
Flessenkamper 2016	+	+	?	?	−	+	?
Gauw 2015	+	+	−	−	−	+	+
Hobbs 1974	?	?	?	?	−	?	?
Kalodiki 2011	+	+	−	−	−	+	+
Kalteis 2015	+	+	−	−	−	+	+
Kolluri 2016	?	?	−	−	−	+	?
Lurie 2005	+	+	−	−	−	+	+

	Random sequence generation (selection bias)	Allocation concealment (selection bias)	Blinding of participants and personnel (performance bias)	Blinding of outcome assessment (detection bias)	Incomplete outcome data (attrition bias)	Selective reporting (reporting bias)	Other bias
Mendes 2016	+	+	+	−	+	−	−
Mozafar 2014	?	?	−	−	?	−	+
Nandhra 2015	?	?	−	−	−	?	?
Pare´s 2010	?	?	?	?	−	+	?
Perala 2005	?	?	?	?	+	−	+
Pronk 2010	?	?	−	−	−	+	+
Rasmussen 2010	+	+	+	−	+	+	+
Rasmussen 2011	+	+	−	−	−	+	+
Rasmussen 2012	+	+	−	−	−	+	+
Rass 2012	+	+	−	−	−	+	+
Rass 2015	+	+	−	−	−	+	+
Rutgers 1994	?	?	−	−	−	+	+
Samuel 2013	?	?	−	−	−	+	?
Sarin 1994	?	?	?	?	+	+	+
Shadid 2012	+	+	?	?	−	+	+
Velden 2015	+	+	?	−	−	+	+
Venermo 2016	?	+	?	?	−	+	+
Yang 2013	?	?	−	−	−	+	?
Yin 2017	+	?	?	−	−	+	+

Figure S2. Network connections of included studies with the available direct comparisons regarding **(A)** successful treatment rate and **(B)** recurrence rate. The details of sample size and trial numbers were presented [CHIVA: Ambulatory Conservative Hemodynamic Management of Varicose Veins].

Figure S2A.

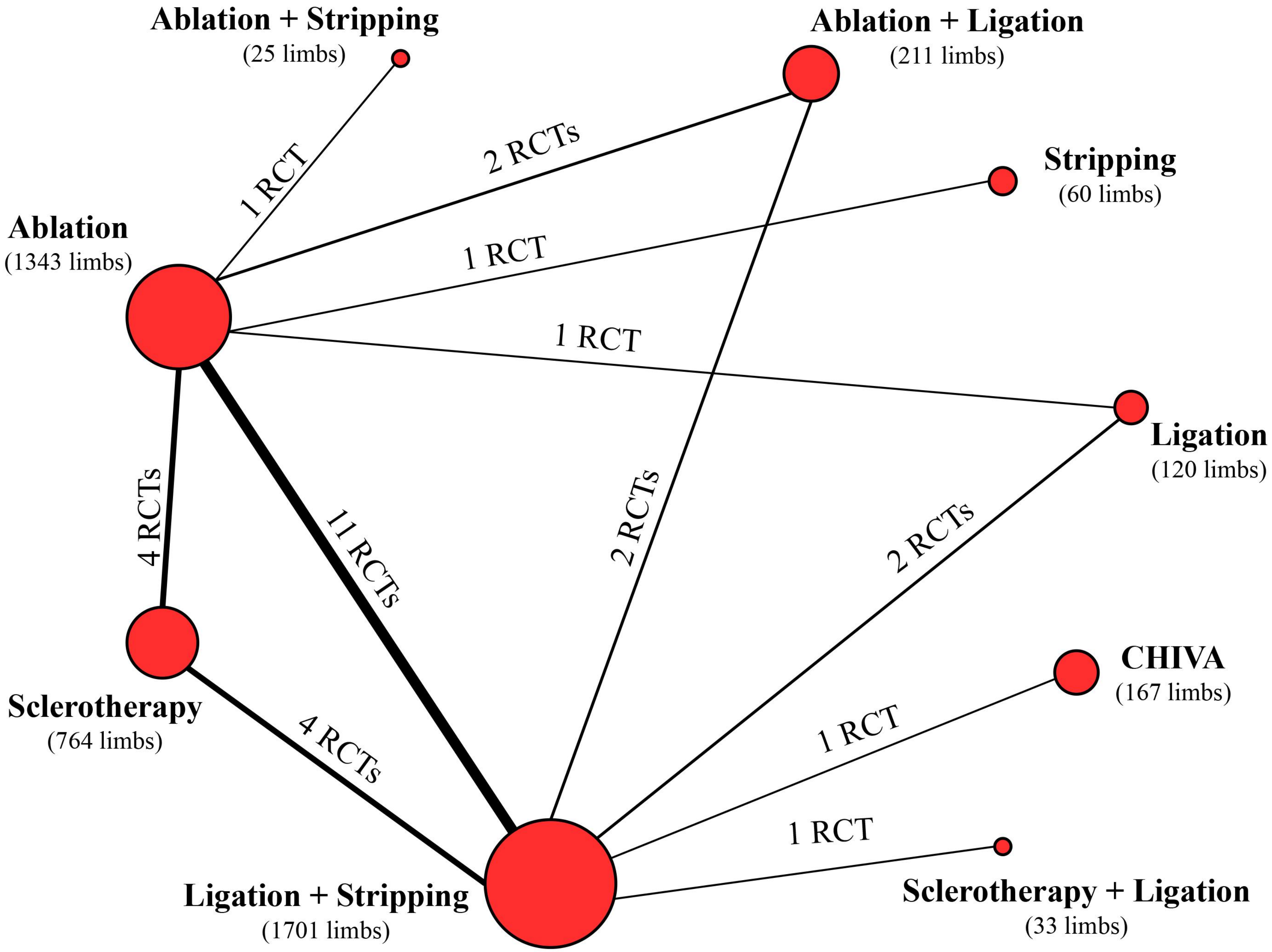

Figure S2B.

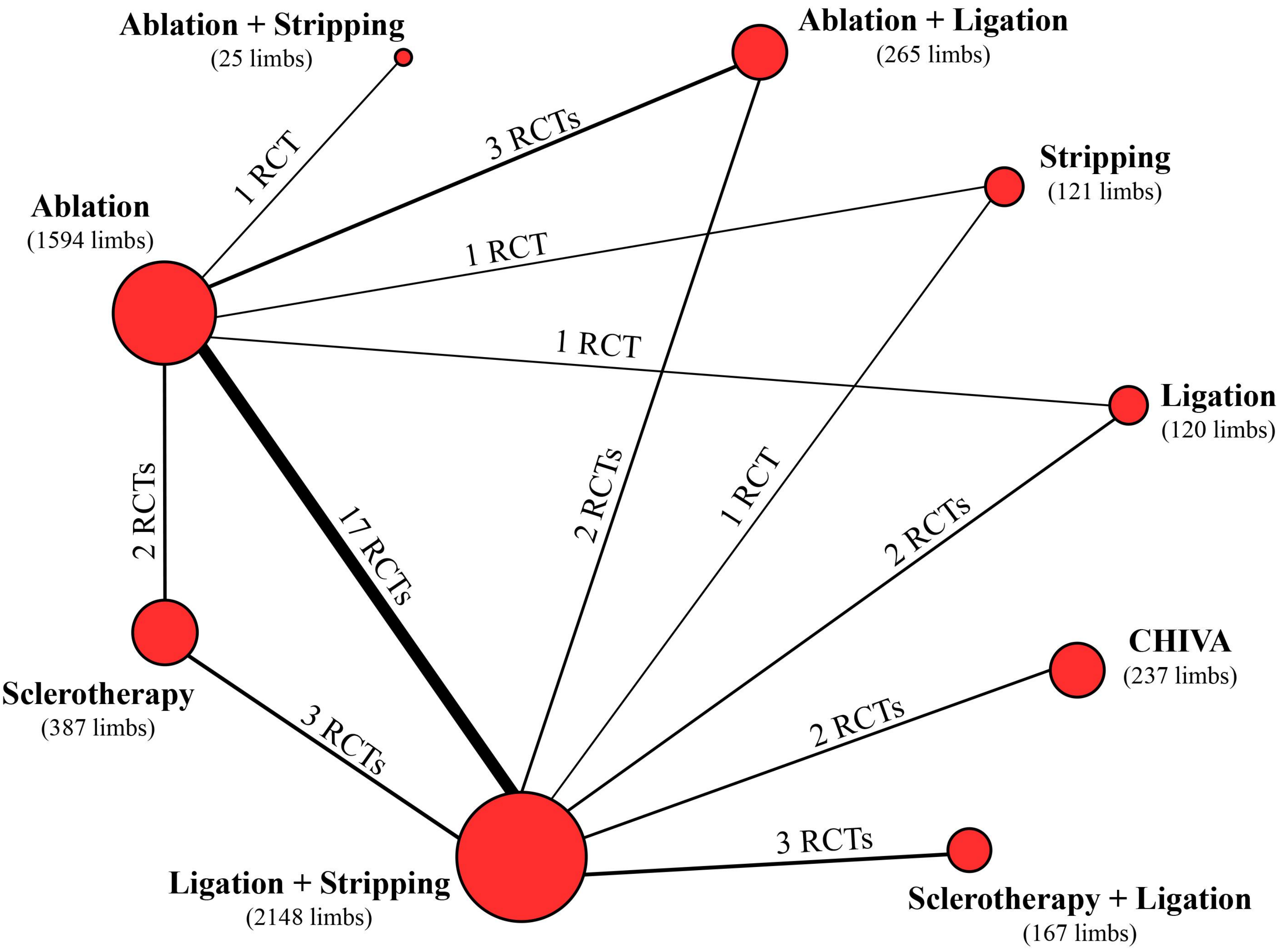

Ablation + Stripping
(25 limbs)
Ablation + Ligation
(265 limbs)
Stripping
(121 limbs)
Ablation
(1594 limbs)
Ligation
(120 limbs)
Sclerotherapy
(387 limbs)
CHIVA
(237 limbs)
Ligation + Stripping
(2148 limbs)
Sclerotherapy + Ligation
(167 limbs)
1 RCT
3 RCTs
1 RCT
1 RCT
2 RCTs
17 RCTs
2 RCTs
1 RCT
2 RCTs
3 RCTs
2 RCTs
3 RCTs

Figure S3. Publication bias of studies regarding **(A)** successful treatment rate and **(B)** recurrence rate was assessed by funnel plots.

Figure S3A.

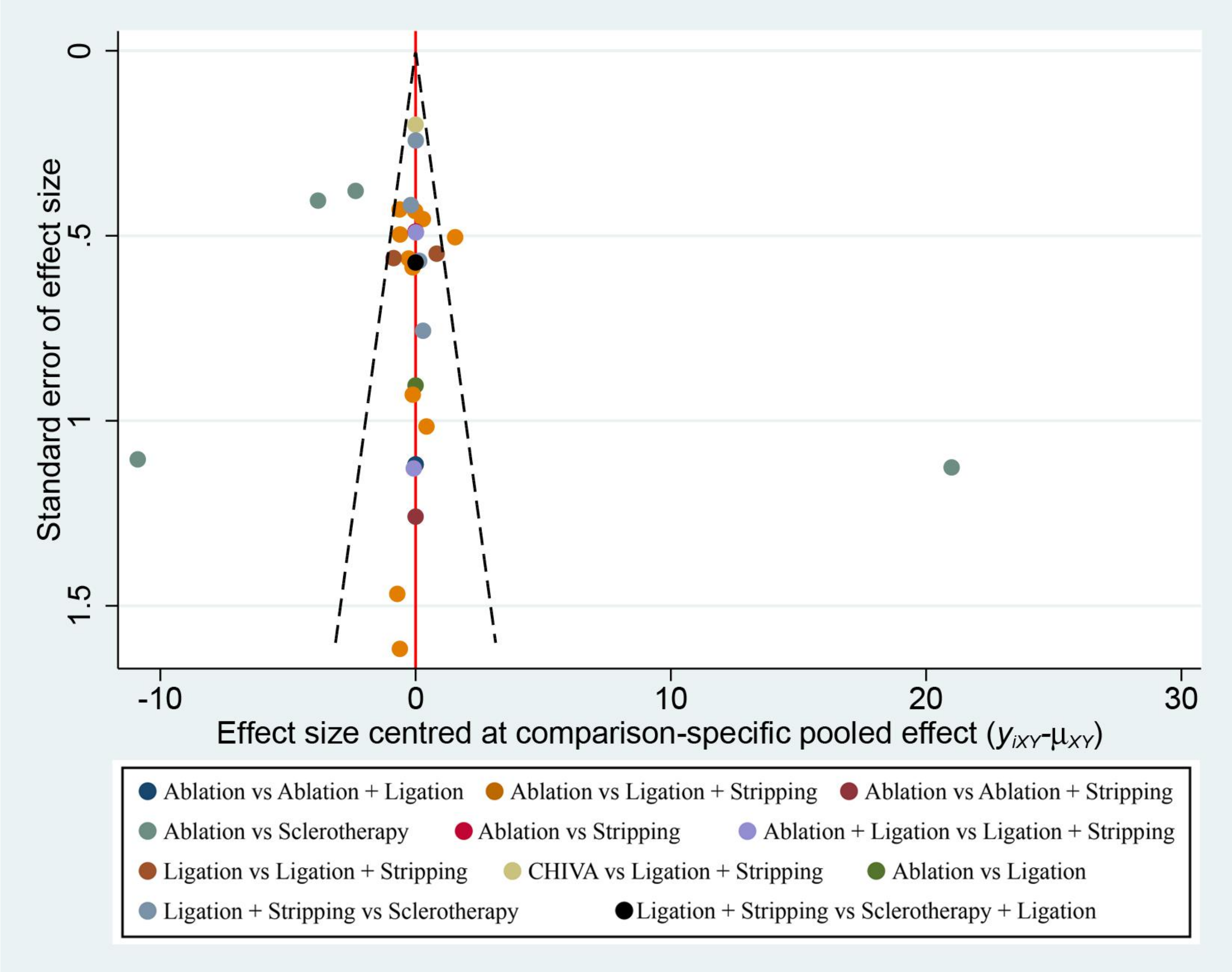

Figure S3B.

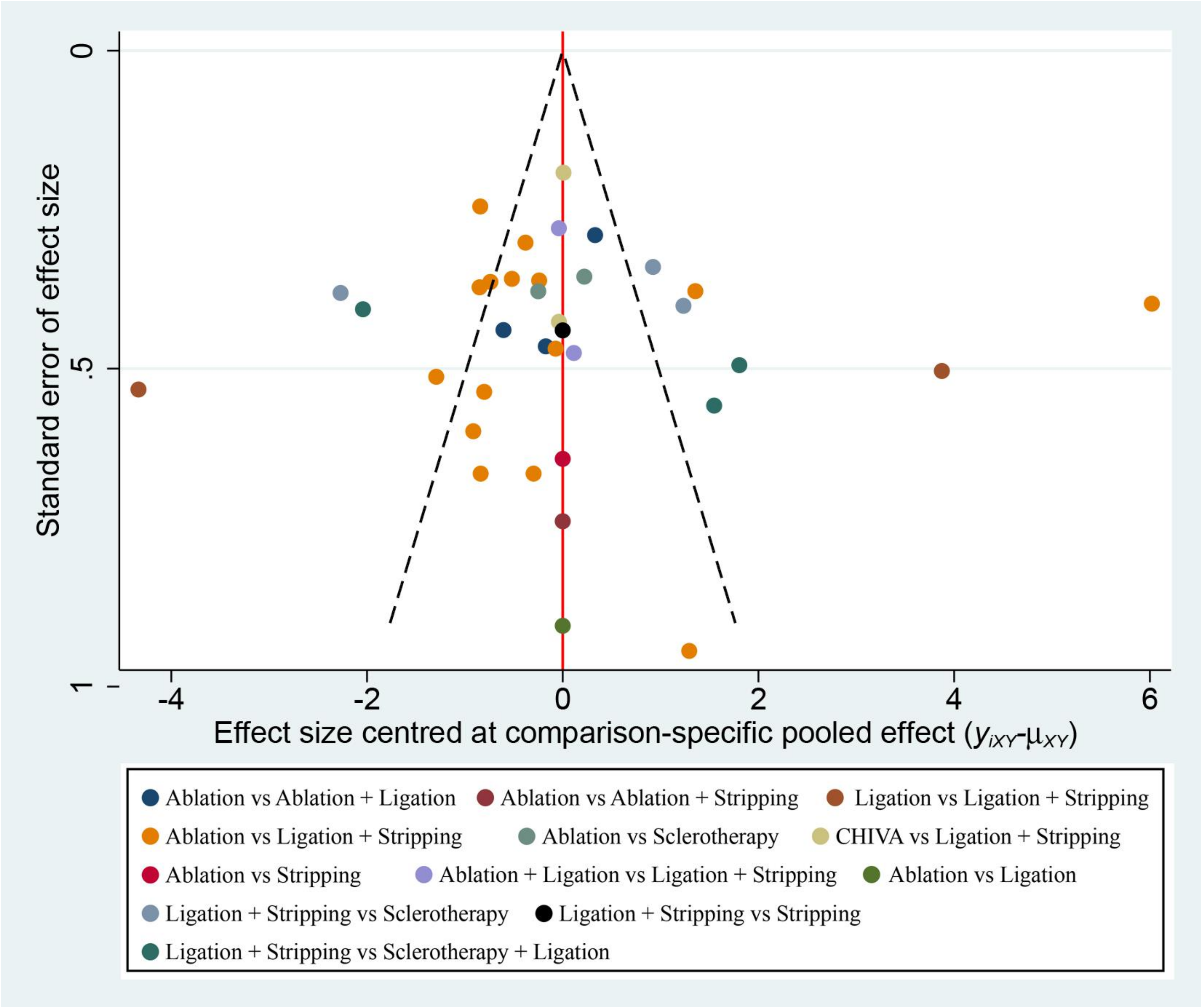

Standard error of effect size
0
.5
1
-4
-2
0
2
4
6
Effect size centred at comparison-specific pooled effect (y_{iXY}-μ_{XY})
Ablation vs Ablation + Ligation
Ablation vs Ablation + Stripping
Ligation vs Ligation + Stripping
Ablation vs Ligation + Stripping
Ablation vs Sclerotherapy
CHIVA vs Ligation + Stripping
Ablation vs Stripping
Ablation + Ligation vs Ligation + Stripping
Ablation vs Ligation
Ligation + Stripping vs Sclerotherapy
Ligation + Stripping vs Stripping
Ligation + Stripping vs Sclerotherapy + Ligation
